# Three Views of a Consultation

**Published:** 1881-08

**Authors:** 


### Three Views of a Consultation.

Bulwer says in one of his novels, in defining a medical consultation, that a consultation is a meeting of physicians in which the counselors agree with the attending physician, and change the treatment.—*Cincinnati Lancet and Clinic.*

A single Doctor like a sculler plies,
The patient lingers and by inches dies.
But two physicians, like a pair of oars,
Waft him with swiftness to the Stygian shores.

—*Jeafferson's Book About Doctors.*

A consultation means that two persons, holding views in some way comparable, adopting principles assimilable, and acting upon lines which may somewhere be made to converge, meet together for the purpose of deciding what is best to be done, under the circumstance, in a case to which these laws and this practice are applicable.—*British Medical Journal.*

